# Activation of LXRα improves cardiac remodeling induced by pulmonary artery hypertension in rats

**DOI:** 10.1038/s41598-017-04640-6

**Published:** 2017-07-21

**Authors:** Yibo Gong, Yifeng Yang, Qin Wu, Ge Gao, Yin Liu, Yaoyao Xiong, Can Huang, Sijie Wu

**Affiliations:** 1 Department of Cardiovascular Surgery, The Second Xiangya Hospital of Central South University, Changsha, China; 2 Faculty of Laboratory Medicine, Xiangya Medical College, Central South University, Changsha, China; 3 Department of Radiology, The Third Xiangya Hospital of Central South University, Changsha, China

## Abstract

Inflammatory factors regulated by NF-κB play a significant role in PAH and myocardial hypertrophy. LXR activation may inhibit myocardial hypertrophy via suppressing inflammatory pathways; it is unknown whether LXR is also involved in PAH-induced myocardial hypertrophy or remodeling. To further explore the protective effect of LXR in PAH-induced cardiac hypertrophy and remodeling, a PAH model was developed, and T0901317, an agonist of LXR, was used to examine the effect of LXR activation. PAH rats demonstrated obvious cardiac hypertrophy and remodeling in the right ventricle, but significant improvement of cardiac hypertrophy and remodeling was observed in PAH rats treated with T0901317. Through RT-PCR, Western blot and ELISA examination, NF-κB, IL-6, TNF-α, and iNOS were found to be significantly reduced in PAH rats treated with T0901317 compared to PAH rats treated with DMSO. Apoptosis was also significantly reduced in PAH rats treated with T0901317. Thus, LXR activation may inhibit PAH-induced cardiac hypertrophy and remodeling by inhibiting NF-κB-mediated inflammatory pathways.

## Introduction

Pulmonary artery hypertension (PAH) often coexists with congenital heart disease, and persistent PAH may lead to ventricular remodeling, right ventricle (RV) enlargement, and right heart failure^1, 2^. However, PAH-induced cardiac hypertrophy and remodeling is largely unstudied. Previous studies have demonstrated that PAH-induced RV enlargement leads to increases in cardiac oxygen consumption and decreases in perfusion, which subsequently accelerates RV remodeling and enlargement. Neuro-hormonal regulation abnormalities, reactive oxygen species (ROS) and reactive nitrogen species (RNS) imbalances, and inflammation may contribute to the disease pathology^3, 5^. Among these conditions, angiotensin II (ATII), endothelin-1 (ET-1), and neuro-hormones promote ROS formation, whereas endogenous nitric oxide (NO) and hemochrome are major resources for RNS formation. Tumor Necrosis Factor-α (TNF-α), interleukin-1 (IL-1), and interleukin-6 (IL-6) are increased in both cardiocytes and the serum of patients with cardiac hypertrophy or failure^6, 7^. Reduced cardiac function in PAH-induced RV hypertrophy may also result from post-apoptotic fibrosis and increases in intercellular fibrin. It has also been recently shown that inhibition of NF-κB may counteract PAH-induced RV hypertrophy^8^.

Liver X Receptors (LXR), a member of the nuclear receptor super-family, was discovered by Willy *et al*. in a liver cDNA database in 1995. Two LXR isoforms, LXRα (NR1H3) and LXRβ (NR1H2), have been identified in mammals and share a high degree of amino acid similarity^9^. Initially, LXRs were considered important regulators of lipid metabolism. In recent years, the role of LXRs in cholesterol metabolism, glycometabolism, bile acid excretion, fatty acid metabolism, immunoregulation, and inflammation has been noted. Recent studies indicate that LXRs regulate the renin-angiotensin-aldosterone system (RAAS)^10–14^. LXRβ is stably expressed in all tissue types, whereas LXRα is mainly expressed in the liver, intestine, and brain. Recently, LXRα was also found to be expressed in the cardiovascular system and may play an important role in this system. Cardiac LXRs may be activated by myocardial infarction^15, 16^, chronic pressure overload^17, 18^, myocarditis^19^, and diabetes^20, 21^. Accumulating evidence implicates intracardiac LXR signaling in the protection against cardiac pathologies involving myocardiocyte hypertrophy and loss, fibrosis, and metabolism dysfunction^17, 18, 22^. The molecular basis for the protective actions of LXRα on pathological hypertrophic growth has been elucidated^18, 23^. LXRα activation inhibits the NF-κB signaling pathway, thus further inhibiting downstream cytokines and expression of target genes such as IL-6, inducible nitric oxide synthase (iNOS), Cyclooxygenase-2 (COX-2), and matrix metalloprotein-9 (MMP-9)^24–26^. Our previous research also showed that LXRα activation may inhibit myocardial hypertrophy induced by lipopolysaccharide (LPS) or Ang II via suppression of the NF-κB pathway in H9C2 cells^18^.

However, it is unknown whether LXRα activation may also improve PAH-induced myocardial hypertrophy or remodeling *in vivo*. No relevant research has been published on this topic to date. In the present study, we examined the effect of LXR activation on myocardial remodeling in a rat PAH model and investigated its possible mechanism.

## Results

### Monocrotatine (MCT) significantly induces PAH and myocardial remodeling in rats

**Figure 1:**
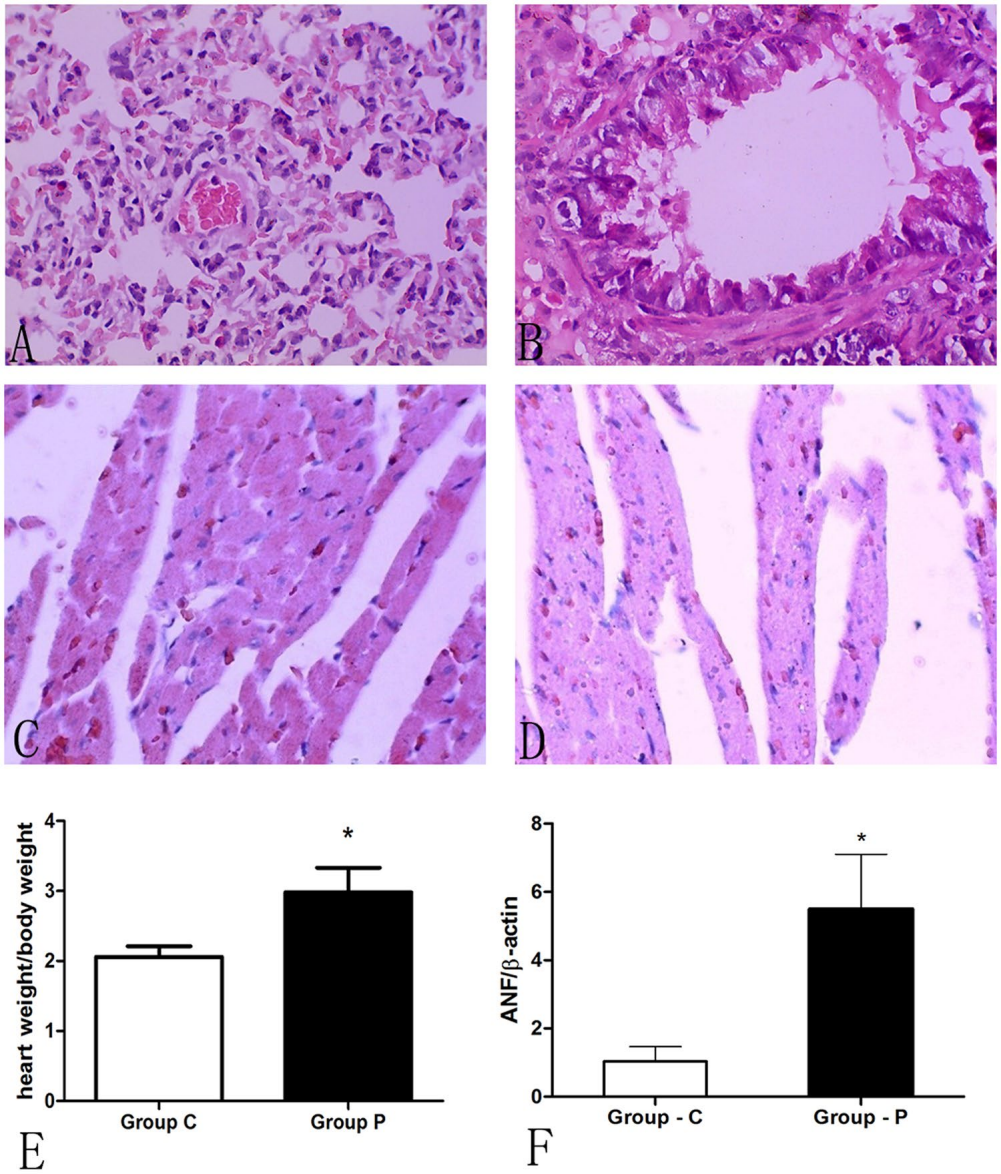
MCT-induced pulmonary artery hypertension and cardiac hypertrophy in SD rats. After administration of MCT (Group P) and saline (Group C) for 28 days, HE staining was performed in the pulmonary artery (**A**,**B**) and right cardiac ventricle (**C**,**D**) (magnification 400X). The heart/weight ratio (**E**) (n = 6, *<0.05) and atrial natriuretic factor (ANF) RT-PCR analysis (**F**) (n = 6, * <0.05) revealed a significantly hypertrophied right ventricle in Group P.

The data were collected on the 28th day after MCT administration, however, respiration frequency increase was observed since the third week in rats with PAH (Group P) relative to control group (Group C). When samples from lung tissues were assessed, lung tissues in Group C exhibited a pink color with good flexibility compared to the sporadic petechiae and decreased flexibility observed in Group P. Megascopic cardiac enlargement was also observed. Hematoxylin-eosin (HE) staining confirmed our suspicions of inflammatory cell invasion, increasing intercellular substance, cell hypertrophy, and apoptosis in the lungs and RV (Fig. 1A–D). Variations were noted in the morphology and heart weight. The ratio of heart weight to body weight (2.983 ± 0.3499 vs. 2.057 ± 0.1548 in Group P and Group C, respectively, Fig. 1E, p < 0.05) was increased in Group P, indicating that cardiac hypotrophy in PAH rats is induced by MCT.

Invasive hemodynamic monitoring (IHM) is considered the gold standard for analysis and directly showed the degree of PAH on day 28 (Table 1). The systolic pulmonary artery pressure (SPAP) (44.57 ± 10.16 mmHg vs. 21.88 ± 4.299 mmHg, p < 0.05), mean pulmonary artery pressure (MPAP) (37.80 ± 8.762 mmHg vs. 17.59 ± 2.866 mmHg, p < 0.05), and mean pulmonary artery pressure/mean arterial pressure (MPAP/MAP) (0.3210 ± 0.049 vs. 0.1497 ± 0.010, p < 0.05) were increased almost 2-fold compared to the normal condition. Blood pressure (BP) and cardiac output (CO) exhibited no differences between the two groups. Ultrasonic cardiogram (UCG) analysis indicated that right heart system expansion occurred in Group P (6.184 ± 0.3632 mm vs. 4.386 ± 0.1491 mm in the RV and 6.433 ± 0.6390 mm vs. 4.512 ± 0.2468 mm in the right atrium (RA), Table 2, p < 0.05). An increase in another important PAH index in clinical practice, aorta/pulmonary artery (AO/PA), was also detected through UCG analysis (1.281 ± 0.1684 vs. 1.057 ± 0.09298, Table 2, p < 0.05). Real-time PCR (RT-PCR) was applied to analyze atrial natriuretic factor (ANF) mRNA levels on day 28 in the two groups (Fig. 1F). ANF was significantly increased in the cardiomyocytes of PAH rats.

**Table 1:** Invasive hemodynamic analysis of rats in the PAH and control groups.

Invasive Hemodynamic Analysis on 28 days		
Parameters	Group C	Group P
SAP(mmHg)	135.7 ± 14.15	136.0 ± 13.36
MAP(mmHg)	117.1 ± 13.80	116.6 ± 11.26
SPAP(mmHg)	21.88 ± 4.299	44.57 ± 10.16*
MPAP(mmHg)	17.59 ± 2.866	37.80 ± 8.762*
MPAP/MAP	0.1497 ± 0.010	0.3210 ± 0.049*
CO	26.26 ± 4.495	27.33 ± 6.132
HR(bmp)	368.8 ± 32.97	372.8 ± 36.58

n = 6, *Compare with Group C, p < 0.05.

**Table 2:** Echocardiography examination results of rats in the PAH and control groups.

Echocardiography Data on 28 days		
Parameters	Group C	Group P
RV(mm)	4.386 ± 0.1491	6.184 ± 0.3632*
RA(mm)	4.512 ± 0.2468	6.433 ± 0.6390*
LV(mm)	4.355 ± 0.5716	4.802 ± 0.8888
LA(mm)	3.539 ± 0.3327	3.301 ± 0.4191
PA /AO	1.057 ± 0.09298	1.281 ± 0.1684*

n = 6, *Compare with Group C, p < 0.05.

In summary, PAH was induced in rats 28 days after MCT administration. Obvious cardiac hypertrophy and remodeling was observed in the right ventricle, with remarkable elevation of ANF expression.

### Agitation of LXRα inhibits cardiac remodeling in PAH rats

**Figure 2:**
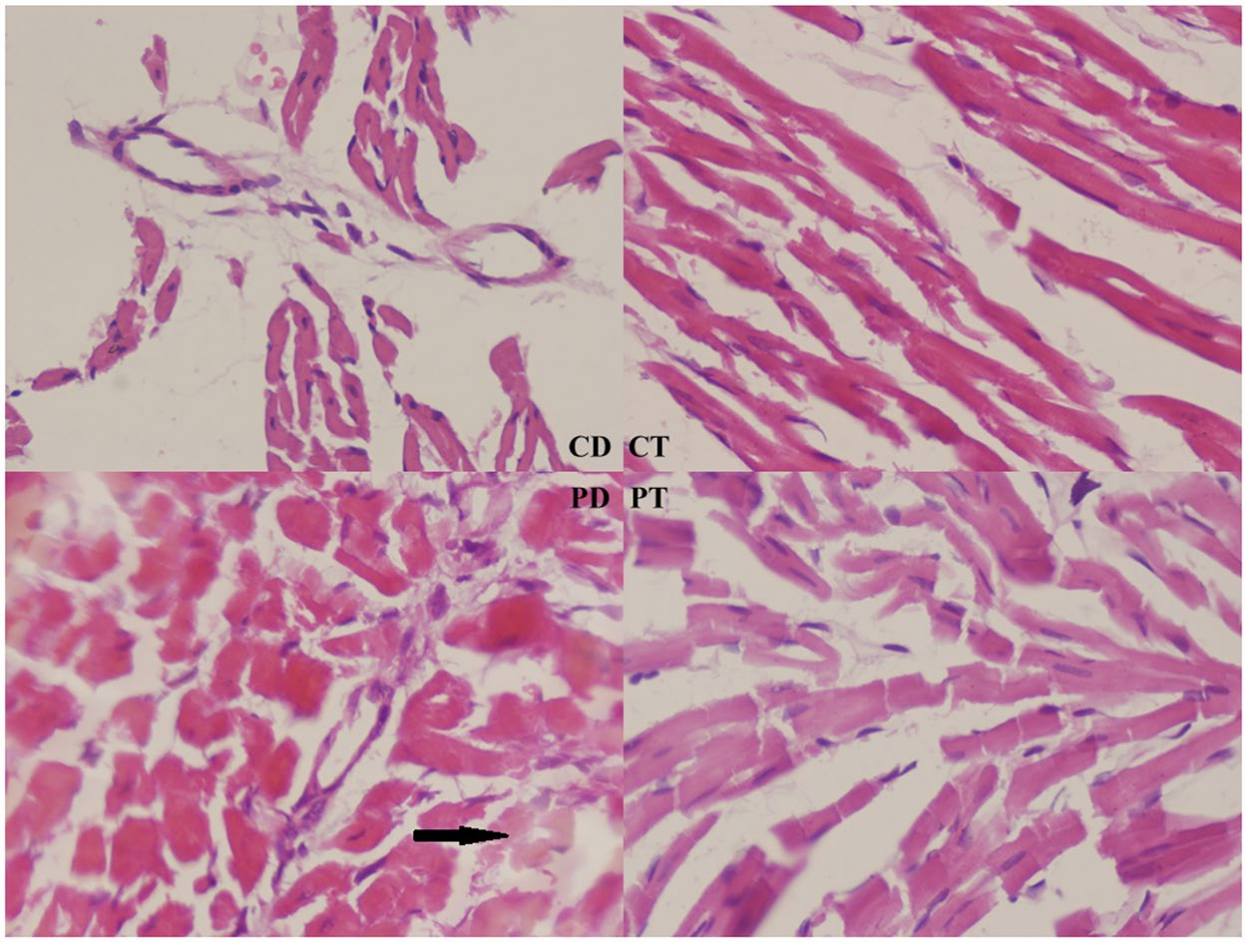
HE staining of rat right ventricular myocardiocytes. CD group rats were treated with normal saline (NS) for 28 days followed by DMSO for 7 days. CT group rats were treated with NS for 28 days followed by T0901317 for 7 days. PD group rats were treated with MCT for 28 days followed by DMSO for 7 days. PT group rats were treated with MCT for 28 days followed by T0901317 for 7 days. The arrow in the PD group indicates myocardiocyte lysis and remodeling (400X). (CD: rats treated with NS + DMSO, CT: rats treated with NS + T0901317, PD: rats treated with MCT + DMSO, PT, rats treated with MCT + T0901317).

**Figure 3:**
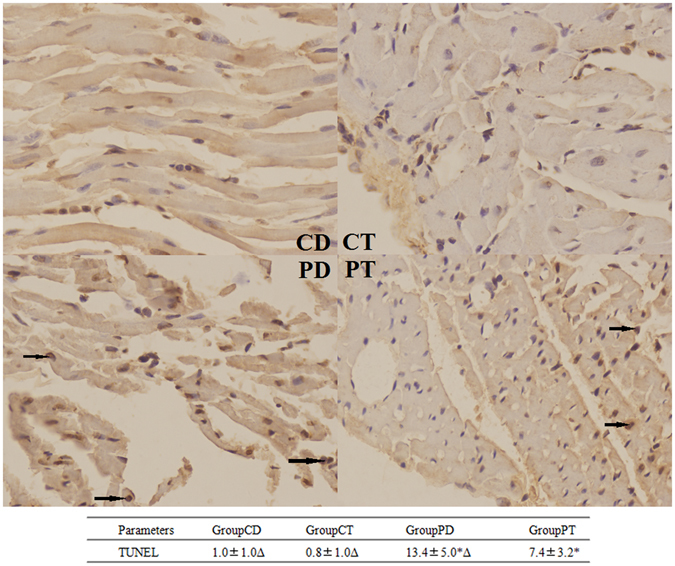
TUNEL staining of the right ventricular myocardium. The arrows indicate apoptotic cells (400X). (CD: rats treated with NS + DMSO, CT: rats treated with NS + T0901317, PD: rats treated with MCT + DMSO, PT, rats treated with MCT + T0901317). (n = 6, *p < 0.05 compared to the CD group, Δ p < 0.05 compared to the PT group).

After treatment of T0901317 or DMSO for 7 days, the four groups (PT, PD, CT, and CD) underwent morphological analysis. No significant differences were noted between the PT and PD groups regarding the heart/weight ratio or echocardiography after statistical analysis (Table 3). Differences were noted in further studies. IHM analysis indicated that the PT group exhibited reduced SPAP (41.21 ± 7.323 vs 50.47 ± 7.220, p < 0.05), MPAP (32.97 ± 5.765 vs. 40.92 ± 5.33, p < 0.05) and MPAP/MAP (0.290 ± 0.035 vs. 0.34 ± 0.023, p < 0.05) (Table 4). HE staining revealed that cardiocytes in both the CD and CT groups were arranged as stripes with relatively large intercellular spaces, which is consistent with typical RV histological characteristics. However, cardiocytes in the PT group exhibited hypertrophy and were slightly mal-arranged with occasional inflammatory cell infiltration, whereas the PD group exhibited the most hypertrophic cardiocytes with obvious apoptosis and massive inflammatory cell infiltration (Fig. 2). TUNEL staining revealed apoptotic cells in the RV of all PAH rats, and no apoptosis was observed in the control group without PAH. After T0901317 administration, apoptosis was significantly decreased compared to that in the PD group (7.4 ± 3.2 vs. 13.4 ± 5.0, p < 0.05) (Fig. 3), suggesting that LXR impeded the development of PAH and myocardial remodeling.

**Table 3:** Echocardiography examination results of rats in the PAH and control groups with or without LXR activation.

Echocardiography Data on 35 days				
Parameters	Group CD	Group CT	Group PD	Group PT
RV(mm)	4.64 ± 0.41	4.71 ± 0.25	6.82 ± 0.36*	6.59 ± 0.54*
RA(mm)	4.77 ± 0.35	4.78 ± 0.26	7.09 ± 0.49*	6.78 ± 0.52*
LV(mm)	4.73 ± 0.38	4.72 ± 0.32	4.82 ± 0.34	5.39 ± 1.1
LA(mm)	3.83 ± 0.35	3.50 ± 0.30	3.70 ± 0.46	3.53 ± 0.50
PA/AO	1.027 ± 0.063	1.008 ± 0.048	1.451 ± 0.134*	1.339 ± 0.096*

n = 6, *Compare with Group CD, p < 0.05.

**Table 4:** Invasive hemodynamic analysis of rats in the PAH and control groups with or without LXR activation.

Invasive Hemodynamic Analysis on 35 days				
Parameters	Group CD	Group CT	Group PD	Group PT
SAP(mmHg)	139.8 ± 15.49	131.6 ± 12.06	140.6 ± 14.21	132.4 ± 12.84
MAP(mmHg)	120.1 ± 16.00	114.1 ± 11.20	120.8 ± 12.84	113.3 ± 9.869
SPAP(mmHg)	23.20 ± 5.143	22.86 ± 3.615	50.47 ± 7.220*	41.21 ± 7.323*Δ
MPAP(mmHg)	18.73 ± 4.15	17.94 ± 2.82	40.92 ± 5.33*	32.97 ± 5.765*Δ
MPAP/MAP	0.16 ± 0.021	0.16 ± 0.026	0.34 ± 0.023*	0.290 ± 0.035*Δ
CO	28.05 ± 4.875	26.22 ± 3.464	26.00 ± 4.004	26.29 ± 3.392

n = 6, *Compare with Group CD, p < 0.05, Δ Compare with Group PD, p < 0.05.

Therefore, compared to PD group, pulmonary pressure in PT group was significantly decreased after T0901317 administration for 1 week, and the cardiac hypertrophy and apoptosis was improved as well.

### LXRα negatively regulates NF-κB, TNF-α, IL-6, and iNOS during myocardial remodeling in the PAH rat model

**Figure 4:**
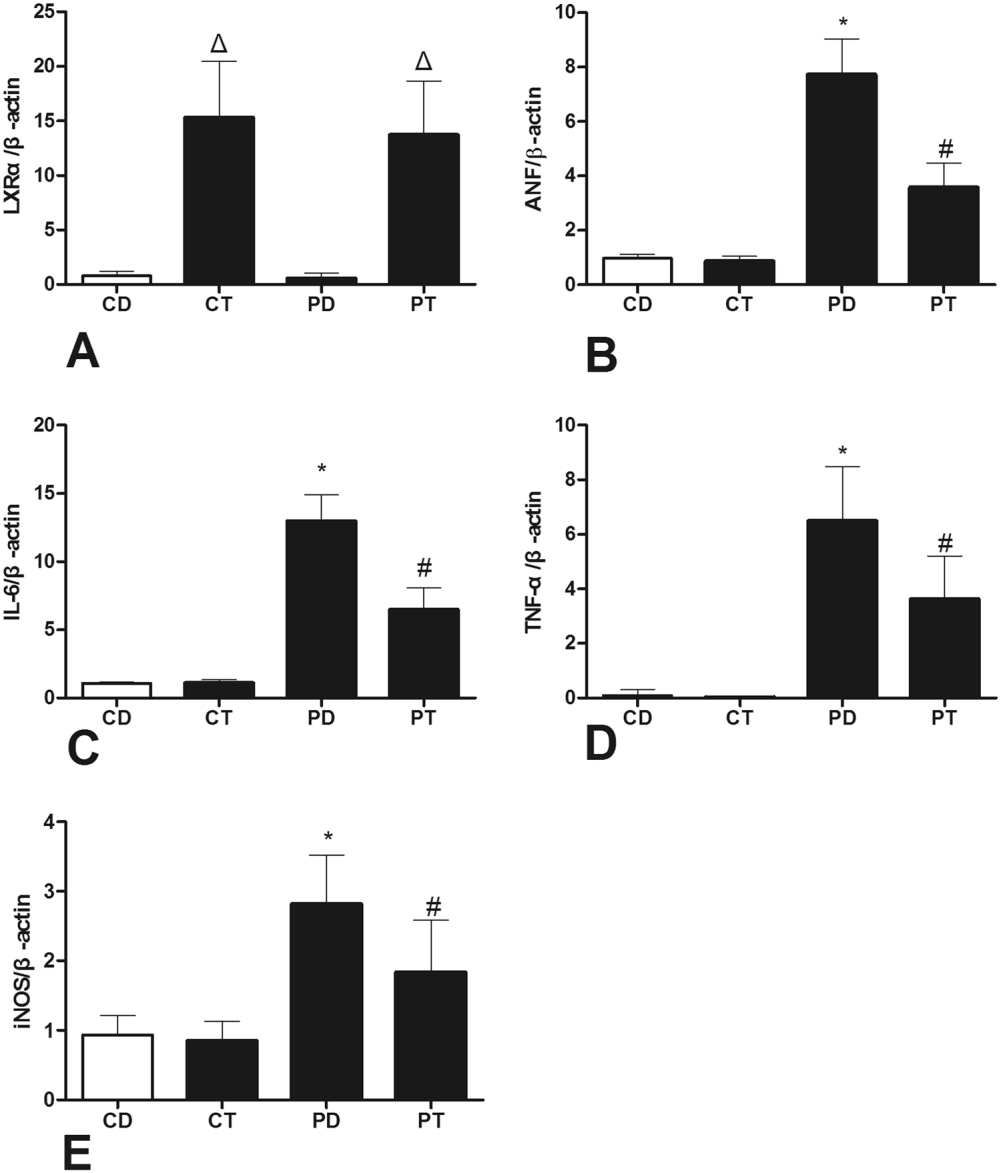
Effects of LXR antagonist administration on RNA activation. Real-time examination revealed mRNA changes of LXRα (**A**), ANF (**B**), TNF-α (**C**), IL-6 (**D**), and iNOS (**E**) expression in rats. RNA was obtained from rat right ventricular myocardiocytes. (CD: rats treated with NS + DMSO, CT: rats treated with NS + T0901317, PD: rats treated with MCT + DMSO, PT, rats treated with MCT + T0901317). (n = 6, Δ: vs CD/PD P < 0.05, *vs CD P < 0.05, ^#^vs PD P < 0.05).

RT-PCR revealed increased expression of LXRα mRNA 7 days after T0901317 treatment in the CT and PT groups (Fig. 4). In addition, cardiac ANF expression was significantly increased in the PAH group compared to the control group and was significantly reduced in the PT group compared to the PD group. TNF-α, IL-6, and iNOS expression exhibited a similar change, as the mRNA expression in the PT group was significantly reduced compared to that in the PD group.

**Figure 5:**
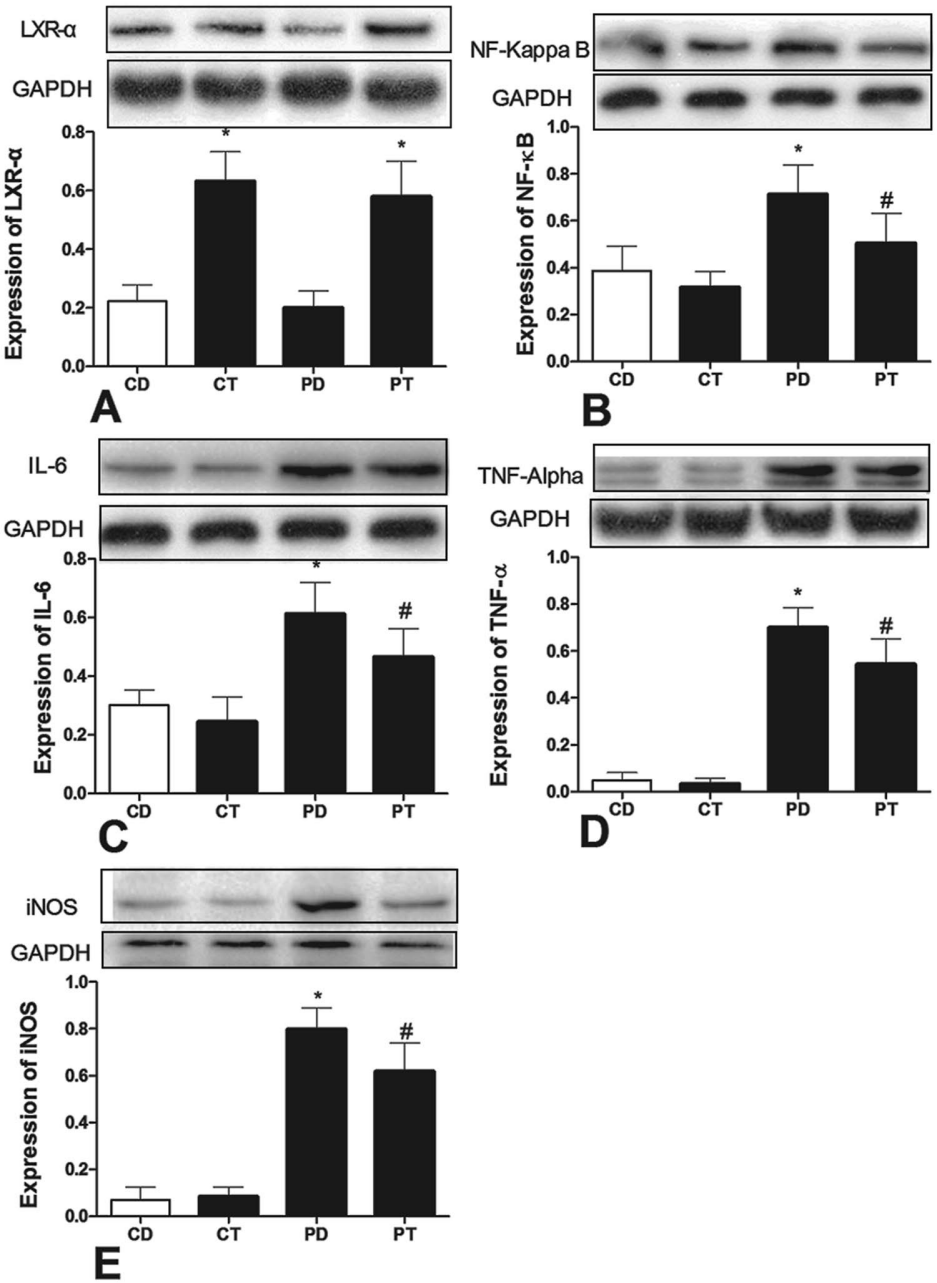
Effects of LXR antagonist administration on protein expression. Western blot examinations revealed changes in LXRα (**A**), NF-κB (**B**), TNF-α (C), IL-6 (**D**), and iNOS (**E**) protein expression in rats. Samples were obtained from rat right ventricular myocardiocytes. (CD: rats treated with NS + DMSO, CT: rats treated with NS + T0901317, PD: rats treated with MCT + DMSO, PT, rats treated with MCT + T0901317). (n = 6, Δ: vs CD/PD P < 0.05, *vs CD P < 0.05, ^#^vs PD P < 0.05).

Western blotting revealed the same trend in protein expression (Fig. 5). T0901317 administration significantly enhanced cardiac LXRα expression. PAH induced increased expression of inflammatory proteins such as NF-κB, IL-6, TNF-α, and iNOS, in cardiocytes. However, the expression of these proteins in cardiocytes was significantly reduced in the PT group compared to that in the PD group.

**Figure 6:**
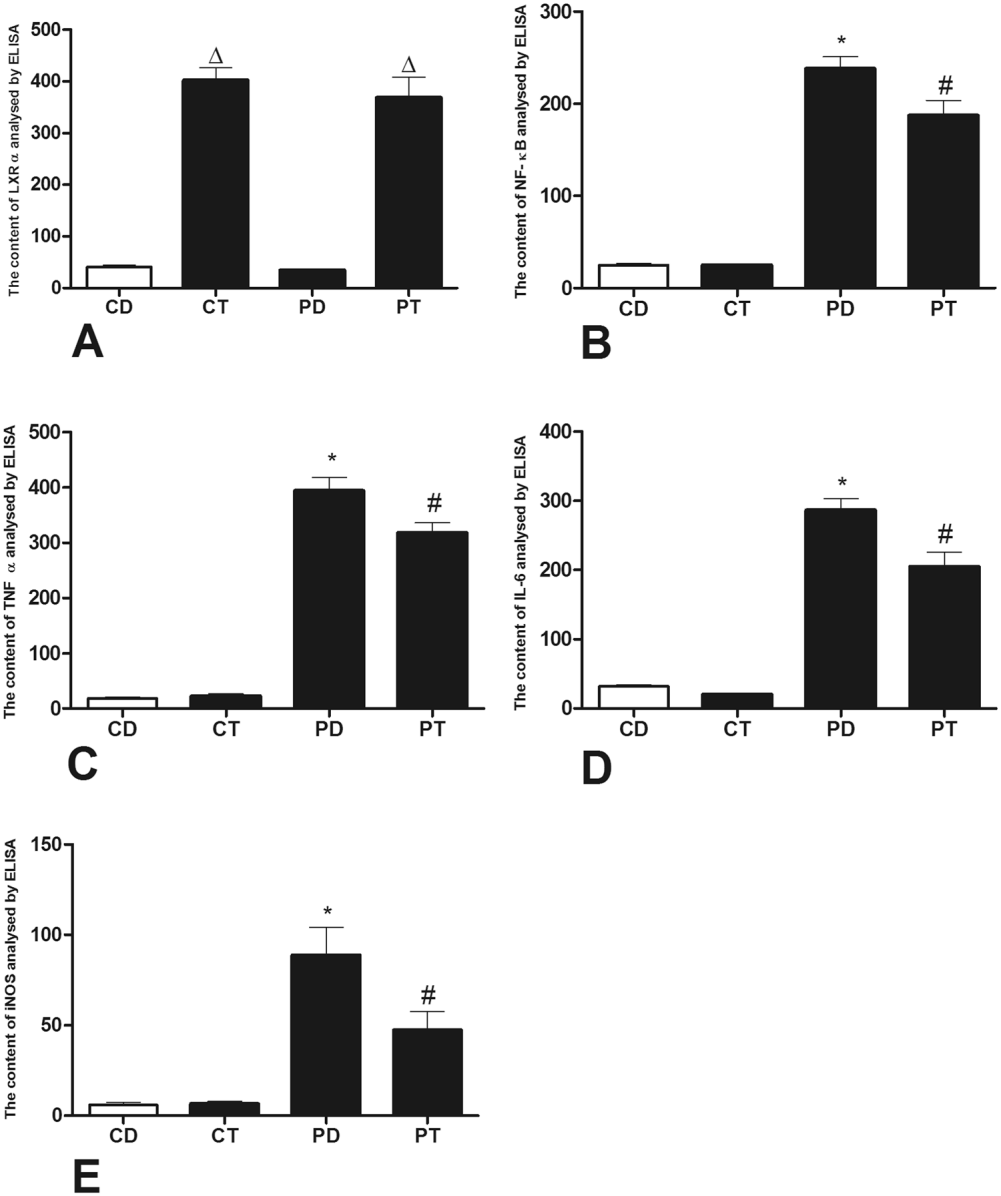
Effects of LXR antagonist administration on protein expression. ELISAs revealed changes in LXRα (**A**), NF-κB (**B**), TNF-α (**C**), IL-6 (**D**), and iNOS (**E**) protein expression in rats. Samples were obtained from rat right ventricular myocardiocytes. (CD: rats treated with NS + DMSO, CT: rats treated with NS + T0901317, PD: rats treated with MCT + DMSO, PT, rats treated with MCT + T0901317). (n = 6, Δ: vs CD/PD P < 0.05, *vs CD P < 0.05, ^#^vs PD P < 0.05).

An ELISA revealed a 4-fold increase in LXR in the PT and CT groups (Fig. 6). NF-κB exhibited a repressed trend with increased LXR expression. An ELISA revealed a significant reduction in NF-κB protein activation in the PT group compared to that in the PD group. NF-κB controls the expression of most inflammatory and apoptosis factors. The down-regulation of NF-κB activity leads to a comprehensive reduction in TNF-α, IL-6 and iNOS following reduced mRNA expression.

Therefore, our study showed T0901317 administration activated LXR expression on myocardiocytes. It also inhibited ANF expression, as well as NF-κb, IL-6, TNF-a, iNOS expression.

## Discussion

PAH is one of the most common causes of cardiac hypertrophy, remodeling, and heart failure, as increased pulmonary pressure leads to post-load increases, ventricular enlargement, and subsequent increases in oxygen consumption. These effects promote cardiocyte fibrosis, cardiac remodeling, and failure. The role of inflammatory signal pathways in cardiocyte hypertrophy and apoptosis has been emphasized in recent studies; however, its role in in PAH-induced cardiac hypertrophy and remodeling remains elusive. Our previous research showed that LXR activation may inhibit the NF-κB pathway in LPS-induced cardiocyte hypertrophy, which may differ from PAH-induced cardiocyte hypertrophy. This study confirmed that LXR activation may also inhibit PAH-induced cardiac hypertrophy and remodeling, and one of the mechanisms may be inhibition of NF-κB -mediated inflammatory and apoptotic pathways.

T0901317, an LXR agonist, activates the expression of both LXRα and LXRβ^27^. LXRβ is stably expressed in multiple tissues, and LXRα is more specifically expressed in the liver, intestine, and heart^28^. Our previous study demonstrated that both LXRα and LXRβ activation may inhibit the NF-κB signaling pathway in cardiocytes. However, LXRα may be more responsive and plays an even broader and more active role in regulating stresses exerted onto the heart. Thus, we examined only LXRα activation in this study.

In our study, at 28 days after MCT treatment, rats exhibited obvious PAH, right heart system expansion, and hypertrophy. These phenomena can be explained by activated inflammation and apoptosis systems and indicate the initiation of myocardial remodeling.

After 7 days of T0901317 administration, the pulmonary pressure in rats mildly decreased, and both the RV size and heart weight ratios exhibited slight decreases on UCG findings. However, the differences were not significant compared to the control group. This result may be affected by the T0901313 administration time. Although macropathological changes in UCG and heart weight results did not reach statistical significance, a histological examination revealed that LXR activation significantly ameliorated PAH-induced cardiac hypertrophy, mal-arrangement, and fibrosis. Thus, we believe that LXR may to some extent inhibit PAH-induced cardiac hypertrophy. As one of the molecular markers of cardiac hypertrophy, ANF was significantly reduced in T0901317-treated PAH rats, confirming the protective effect of LXR activation. In addition, LXR activation also ameliorated cardiac apoptosis in the RV, which may contribute to its influence on cardiac remodeling.

Inflammation and apoptosis factors, such as iNOS, COX-2, IL-6, IL-1, and MMP-9, were induced by LPS and reduced by LXR^24–26^. In this study, we measured NF-κB, TNF-α, IL-6, and iNOS expression levels in right ventricular tissues and noticed a significant increase in these classic inflammation and apoptosis factors. The activation of NF-κB affects myocardial remodeling. Active NF-κB is transferred into the cell nucleus through IκB degradation^29^, and expression of genes that influence the production level of downstream inflammation and apoptosis factors is up-regulated. T0901317 suppressed NF-κB activity in the heart in PAH rats, suggesting that LXRs inhibit cardiac hypertrophy and inflammation via the NF-κB signaling pathway. Our experiment showed that NF-κB and downstream IL-6 and TNF-α were significantly down-regulated. However, cardiac apoptosis and iNOS expression were reduced. Thus, LXR activation may inhibit cardiac fibrosis and remodeling by inhibiting the apoptotic pathway. Further experiments are warranted to confirm this finding.

As indicated in recent literature, LXR may inhibit toll-like receptor-4 (TLR-4) signaling in macrophages^30^. However, significant TLR-4 expression was not observed in this study and in our previous *in vitro* study. Thus, we assume that the TLR-4 pathway may not be involved in LXR-mediated inflammatory pathways. In addition, although AT1 is inhibited by LXR activation in smooth muscle^31^, we did not observe any significant difference in AT1 expression in PAH cardiocytes. Similar results were noted in our previous *in vitro* experiment. However, these findings await further confirmation.

Recent literature revealed that ROS might function as key mediators of mechanotransduction during both physiological adaptation to mechanical load and maladaptive remodeling of the heart^32^. LXRs have also been shown to regulate cell survival through inhibition of ROS production and oxidative stress^33, 34^, as well as prevent apoptosis induced by hyperglycemia^35^ and diabetes^21^. Our study also examined ROS expression change after LXR activation, but no stable results were obtained (data not shown). As sampling variation should be considered, further research is warranted to confirm whether ROS signaling pathway is involved in LXR mediated improvement of right ventricular remodeling.

In summary, PAH may lead to RV hypertrophy and remodeling. The NF-κB-mediated inflammatory pathway may be a mechanism of inducing inflammatory and apoptotic cytokine expression, leading to subsequent cardiac hypertrophy and increased apoptosis and cardiac fibrosis. LXR activation may inhibit NF-κB and its downstream inflammatory cytokines, such as IL-T, TNF-α, and iNOS, which could improve PAH-induced cardiac hypertrophy and remodeling.

## Methods

### Animals and animal treatments

One hundred healthy SD rats were randomly divided into two groups (the control group (Group C) and PAH group (Group P), 50/group). Then, 60 mg/kg MCT was administered by intraperitoneal injection to rats in Group P^36^. Group C rats were administered the same dose of NS. After 28 days, 80 rats (40 randomly selected from each group) were separated into 4 groups. Group C was divided into the CD group, in which rats were treated with 10 mg/kg*d DMSO, and the CT group, in which rats were treated with the LXR agonist T0901317 (10 mg/kg*d) by intragastric administration for 7 days^37, 38^. In a similar manner, Group P was divided into the PD group, in which rats were treated with DMSO, and the PT group, in which rats were treated with the same dose of T0901317. Rats were maintained on a 12-h light/dark cycle in temperature-controlled rooms and had ad libitum access to water and standard laboratory chow. All experimental procedures were approved by the Institutional Animal Care and Use Committee of the Central South University, and performed in accordance with relevant guidelines and regulations.

### UCG detection

UCGs were used to describe morphological changes at 28 and 35 days. Rats were narcotized by 10% chloral hydrate at a dose of 3 mg/kg. Data from the RV and the RA were collected from a four-chamber view, and data from the left ventricle (LV), left atrium (LA), and aorta (AO) were obtained from the long axis. Pulmonary artery (PA) data were derived from the horizontal shaft axis, and CO was obtained using the m-echo mode.

### Invasive hemodynamic monitoring

As the gold standard of PAH, IHM was used to estimate the level of PAH at 28 and 35 days. SD rats underwent basic anesthesia and were supported using a ventilator. While sustaining sufentanil and vecuronium support, we opened the chest and pericardium and directly detected the PA pressure with a PE10 catheter imbedded into the PA through the right ventricular outflow tract (RVOT). BP and central venous pressure (CVP) were monitored at the arteria femoralis and jugular vein, respectively. All data were managed using PowerLab™.

### Gene expression analysis

The TRIZOL reagent from Invitrogen was used to isolate total RNA. RT-PCR was performed using the Prime Script RT reagent kit with gDNA Eraser (Takara, Japan). Quantitative real-time PCR (qRT-PCR) was performed using the All-in-one TM qPCR Mix kit (Gene Copenien, USA). All primers for RT-PCR were synthesized at Sangon (Shanghai, China). RT-PCR results from each gene/primer pair were normalized to the results of β-actin and compared across conditions. The primer sequences used for qPCR amplification were as follows: LXRα: forward- AGG GCT GCA AGG GAT TCT TC, reverse- CCT CGA TCG CAG AGG TCT TC; IL-6: forward-ACA GTG CAT CAT CGC TGT TC, reverse-CCG GAG AGG AGA CTT CAC AG; TNF-α: forward-ACT CCC AGA AAA GCA AGC AA, reverse-CGA GCA GGA ATG AGA AGA GG; ANF: forward-CAC CTT GGA GTT CAC CCA GT, reverse-ACC ACT CGT ACT TGG GAT GC; iNOS: forward-CAC CTT GGA GTT CAC CCA GT, reverse-ACC ACT CGT ACT TGG GAT GC; β-actin: forward-CAT CCT GCG TCT GGA CCT GG, reverse-TAA TGT CAC GCA CGA TTT CC.

### Western blot analysis

Nuclear and cytosolic proteins were extracted from RV tissue samples using a nuclear protein extraction kit (Pierce). Protein samples were subjected to SDS-PAGE and Western blotting using the Bio-Rad Image Lab program. Antibodies were obtained from commercial sources. LXRα, NF-κB p65, IL-6, TNF-α, and iNOS were obtained from Abcam, Inc. GAPDH was obtained from Xianzhi Biotechnology, China.

### Paraffin sections and HE staining

After gradient dehydration at room temperature, myocardial tissues were embedded in paraffin and cooled in a 20 °C refrigerator for 1 hour. Tissue sections were cut at 5 microns, bathed in 45 °C water, and dried for preservation. After the sections were dried, hematoxylin was used for nuclei staining, and eosin was used to stain the cytoplasm after dewaxing and hydration. In the last step, the sections were sealed with neutral gum for observation.

### TUNEL staining

All slides were incubated in dUTP labeled with 50 μl TdT and 450 μl fluorescein. The reaction was performed for 1 hour in the dark. For the negative control, only dUTP and 50 μl fluorescein were added. For the positive control, dUTP in combination with a 50 μl TdT and 450 μl fluorescein mixture was added after DNase treatment. After PBS washing, the converter-POD was reacted for 30 min at 37 °C. Finally, DAB staining was performed, and hematoxylin was used to stain the nucleus.

### Statistical analysis

A t-test was used to analyze the UCG, invasive hemodynamic analysis, myocardial weight, RT-PCR, and Western blot data. One-way ANOVA was used to compare data among the four groups. A value of p < 0.05 was considered significant. All graphs were processed with GraphPad Prism 5.
